# Participation rates, response bias and response behaviours in the community survey of the Swiss Spinal Cord Injury Cohort Study (SwiSCI)

**DOI:** 10.1186/s12874-015-0076-0

**Published:** 2015-10-08

**Authors:** Christine Fekete, Wolfgang Segerer, Armin Gemperli, Martin WG Brinkhof, for the SwiSCI Study Group

**Affiliations:** Swiss Paraplegic Research, Guido A. Zäch Strasse 4, 6207 Nottwil, Switzerland; Department of Health Sciences and Health Policy, University of Lucerne, Frohburgstrasse 3, P.O. Box 4466, 6002 Lucerne, Switzerland

**Keywords:** Survey methodology, Community survey, Spinal cord injury, Response rates, Participation rates, Non-respondent, Non-response bias

## Abstract

**Background:**

Surveying persons with disabilities is challenging, as targeted subjects may experience specific barriers to survey participation. Here we report on participation rates and response behaviour in a community survey of people with spinal cord injury (SCI) in Switzerland. The cross-sectional survey was implemented as part of the Swiss Spinal Cord Injury Cohort Study (SwiSCI) and represents the largest population-based SCI survey in Europe including nearly 2000 persons. Design features to enhance participation rates included the division of the questionnaire volume over three successive modules; recurrent and mixed-mode reminding of non-responders; and mixed-mode options for response.

**Methods:**

We describe participation rates of the SwiSCI community survey (absolute and cumulative cooperation, contact, response, and attrition rates) and report on response rates in relation to recruitment efforts. Potential non-response bias and the association between responders’ characteristics and response behaviour (response speed: reminding until participation; response mode: paper-pencil vs. online completion) were assessed using regression modelling.

**Results:**

Over the successive modules, absolute response rates were 61.1, 80.6 and 87.3 % which resulted in cumulative response rates of 49.3 and 42.6 % for the second and third modules. Written reminders effectively increased response rates, with the first reminder showing the largest impact. Telephone reminders, partly with direct telephone interviewing, enhanced response rate to the first module, but were essentially redundant in subsequent modules. Non-response to the main module was related to current age, membership of Swiss Paraplegic Association (SPA) and time since injury, but not to gender, lesion level and preferred language of response. Response speed increased with household income, but was not associated to other sociodemographic factors, lesion characteristics or health indicators. We found significant associations between online completion and male gender, younger age, higher education, higher income, SPA membership, tetraplegia, longer time since injury, higher quality of life, and more participation restrictions.

**Conclusion:**

In this sample with little non-response bias, recurrent and mixed-mode reminding and mixed-mode options for response were key features of optimizing survey design.

## Background

Epidemiological studies that aim to survey persons with disabilities are challenging [1, 2]. As compared to unaffected or able-bodied persons, several factors may render subjects more reluctant to survey response. Similar to the overall engagement in social and community life, survey participation may be restrained as a result of bodily impairments and contextual barriers [1–3]. Also, certain disability populations may face intensive, repeated and uncoordinated surveillance from diverse addressors such as research institutions, health care providers or insurance companies. This over-surveying may predispose subjects to develop a general reluctance to study participation, particularly if past commitments were experienced as unrewarding [4]. Although it is assumed that living with a disability affects survey participation, other factors such as personal motivation, personal interests or attitudes towards research may also determine study participation [5, 6].

Optimal survey design for populations with impairments or disabilities should acknowledge specific challenges and implement methodology that assists in reducing barriers to participation [1, 2]. Yet, most of the experimental and observational evidence on response rates and response behaviour is derived from studies on able-bodied populations [7]. The present study provides evidence from a disability-afflicted population, based on a community survey that was performed in the context of spinal cord injury (SCI), as part of the Swiss Spinal Cord Injury Cohort Study (SwiSCI) [8]. SCI has a far-reaching impact on a person’s functioning and health as affected persons suffer from a loss of sensory and motor function below the lesion level [9]. The SwiSCI population has developed as the largest national cohort on persons with SCI in Europe and provides a comprehensive database to evaluate functioning, disability and health.

Critical features of the SwiSCI survey design with respect to participation rates included 1) the division of the questionnaire volume over three successive modules, 2) recurrent and mixed-mode reminding of non-responders by written and telephone notices, and 3) mixed-mode options for response including paper-pencil questionnaire, online questionnaire, or telephone interview [10]. An in-depth reporting of the recruitment procedure and recruitment outcomes of the SwiSCI community survey is an essential basis for future reporting of study results. Participation rates and non-response bias are two crucial indicators of the survey quality [4]. Although participation rates do not reflect a potential non-response bias inherent in a sample [11], the transparent reporting of participation rate calculation is critical as there are different definitions and approaches of calculation [4, 12–14]. Furthermore, the evaluation of response behaviour according to subjects characteristics and the effectiveness of recruitment efforts in terms of achieved response rates might deliver important information for the planning of recruitment strategies in upcoming community surveys targeting persons with disabilities.

The objective of this study is to report on participation rates, non-response bias and response behaviour in the SwiSCI community survey. More specifically, we aim 1) to describe participation rates by reporting on absolute and cumulative cooperation, contact, response, and attrition rates, 2) to illustrate response rates in relation to recruitment efforts, 3) to compare basic characteristics of respondents and non-respondents in order to evaluate non-response bias, and 4) to describe response behaviour (speed and mode) in relation to responders’ characteristics (sociodemographics, lesion characteristics and health indicators).

## Methods

### Design

The recruitment of the SwiSCI community survey was conducted between Sept 2011 and March 2013. The survey 2011–2013 contained three subsequent modules that were sent out with an interval of about 3 months: 1) the first module (“Starter Module”), a brief 19-item questionnaire on basic socio-demographics, lesion characteristics and the care situation; 2) the second and main module (“Basic Module”), a 124-item questionnaire comprising detailed information on functioning, health, environmental and personal factors; and 3) the third module, three thematically different specific modules to which participants of the second module were randomly assigned. The specific modules included the “Psychological Personal Factors and Health Behavior Module” (PPF-HB) module (186 items), the “Work” module (79 items), and the “Health Services Research” (HSR) module (202 items). In case of the Work module, only employable persons (<65 years) were eligible [8]. Subjects received a paper-pencil version of the questionnaire in all mailings (invitation letter, first and second reminder). On the title page of each questionnaire, the login data for online completion was given. In addition, the toll-free SwiSCI-helpline number as well as the SwiSCI-email address was provided with the notice that participants could contact the study center in case they needed assistance in questionnaire completion.

The process of determining what to measure in the SwiSCI community survey was guided by the Core Sets for SCI of the International Classification of Functioning, Disability and Health (ICF) [15]. Core Sets are lists of generally agreed-upon categories from the entire ICF classification that capture most relevant ICF categories for specific diseases (www.icf-core-sets.org). Additionally to the Generic Set including basic ‘must have’ information for all disabling conditions (www.icf-research-branch.org), all ICF categories defined in the Brief ICF Core Sets for SCI [16, 17] were assessed in the first and the second module. Brief ICF Core Sets include as few categories as possible to be practical, but as many as necessary to be comprehensive in describing the typical spectrum of functional problems in persons with SCI [16, 17]. The topics of the thematically specific third modules have been decided by extensive expert discussions.

### Sample

The SwiSCI community survey aimed at including all persons aged over 16 years with traumatic and non-traumatic SCI living in Switzerland. Due to a lack of central registries on persons with SCI in Switzerland, the study population was established based on records from three specialized rehabilitation centres (REHAB Basel; Swiss Paraplegic Center, Nottwil; Clinique Romande de Réadaptation, Sion) and two national associations for persons with SCI (Parahelp; Swiss Paraplegic Association) [8]. In total, 3807 persons were identified and invited for survey participation. Thereof, 663 persons were non-eligible, resulting in an eligible population of 3144 persons. From the total of 3144 eligible persons, 1922 participated in the first module, 845 refused participation and 377 could not be contacted (details on response status across all modules can be found in the Results section, Table 3). As cooperating institutions were not entitled to share postal addresses from identified subjects, the first module was sent out through these institutions with a request for written consent to address information access by the SwiSCI study center for future correspondence. In total, 100 persons refused consent on address access and further correspondence and data collection was effectuated by the collaborating institutions.

In order to establish a cohort of individuals with acquired SCI in stable conditions, individuals with congenital conditions leading to SCI (e.g. spina bifida), new SCI in the context of palliative care, neurodegenerative disorders (e.g. multiple sclerosis, amyotrophic lateral sclerosis), Guillain-Barré syndrome, and locked-in syndrome were excluded. The SwiSCI study has been approved by the Ethics Committee of the Canton of Lucerne and all participants gave written informed consent. The SwiSCI community survey has been performed in accordance with the ethical standards laid down in the 1964 Declaration of Helsinki and its later amendments.

### Recruitment

A catchy study name and a logo were developed and applied on all study materials to create project identity. A toll-free help line and an address (web and postal) to contact study staff were provided. A multi-mode approach was used for recruitment. The invitational letter was sent through the collaborating institutions and included a motivation letter signed by the Directors of the collaborating institutions and the Director of Swiss Paraplegic Research as the host of the SwiSCI study; the study information; two copies of the informed consent form; the paper-pencil questionnaire of the first module; and a pre-printed, addressed and post-paid envelope to return the questionnaire. In the invitational letter, an incentive (USB-stick and lanyard keychain with SwiSCI-logo) within the mailing of the second module was announced.

**Fig. 1:**
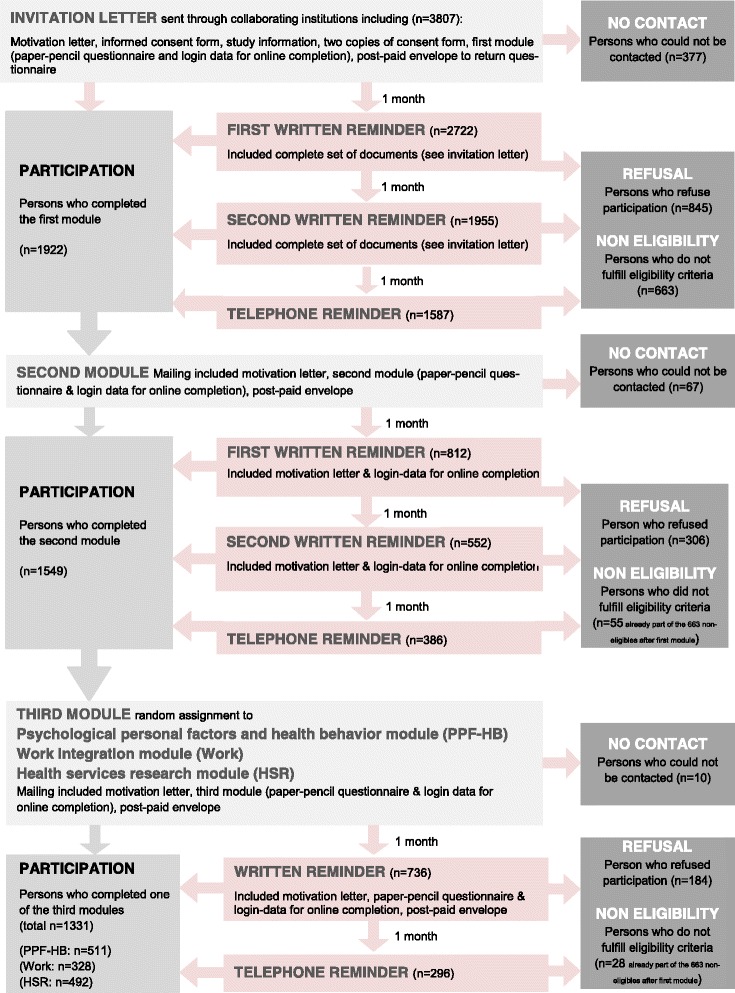
Recruitment and reminder strategy of the SwiSCI community survey

For the first two modules, the same reminder schedule was applied. Persons who did not respond to the invitation letter were re-contacted by up to two written reminders with the interval of a month. Non-responders were finally contacted by telephone a month after the last written reminder to get a final decision on participation or refusal. With the exception that no second reminder was sent, the same procedure was applied for the third modules. In case the reminder and the participants response were timely very close, it was difficult to evaluate whether the response was triggered by the reminder or not. We therefore defined the rule that for postal responses >2 days after the send out of a reminder was classified as having received a reminder, for online responses >1 day and for telephone interviews 0 days. Study material sent with the different mailings varied by module (Fig. 1).

### Measures

#### Response behaviour

Response speed and mode were used as two variables to describe participants’ response behaviour. Response speed was defined as the effort needed until a person’s survey participation (return of the completed questionnaire after the invitational letter, the first, the second or the telephone reminder). Response mode was operationalized by the information on how participants completed the questionnaires (3-categorical variable: paper-pencil, online, telephone interview).

#### Sociodemographic characteristics

We used age in years, gender, employment status (employed vs. not employed), living arrangement (alone vs. with others), partnership (partner vs. no partner), language region (German, French, Italian) and membership of Swiss Paraplegic Association (SPA; yes vs. no) as sociodemographic information. Additionally, education and income were used as indicators for individual-level socioeconomic position. Education was classified according to the International Standard Classification of Education as total years of formal education, combining school and vocational training [18]. Income was measured by net equivalent household income, including information on disposable income, household size and number of adults and children according to guidelines of the Organisation for Economic Co-operation and Development (OECD) [19].

#### Lesion characteristics

Variables on lesion characteristics included lesion level (para- vs. tetraplegia), extent of lesion (complete vs. incomplete), aetiology (traumatic vs. non-traumatic), and years since injury.

#### Indicators of health and functioning

Information on the prevalence and severity of secondary conditions during the past 3 months was collected using the Spinal Cord Injury Secondary Conditions Scale (SCI-SCS, 13 items on a 4-point Likert scale, range 0–39, higher scores indicating higher prevalence and severity) [20]. To measure mental health, we used the 5-item Mental Health Subscale of the 36-item Short Form Health Survey (SF-36) and computed a sum score ranging from 0 to 100 with higher scores indicating better mental health [21]. Activity and participation was measured using the 11-item subscale on participation restrictions of the Utrecht Scale for Evaluation of Rehabilitation-Participation (USER-participation) [22]. A sum score on the number of 4-categorical items that were applicable to a person was built and converted into a score ranging from 0 to 100 (higher scores indicating lower participation restrictions) [22]. Quality of life was assessed by a single item rated on a 5-point Likert-scale ranging from 0 (very poor) to 4 (very good).

#### Variables for comparison between respondents and non-respondents

To evaluate determinants for response and non-response, the following variables were available for all persons that were initially invited to participate in the survey: current age, gender, preferred language (German, French, Italian), membership of SPA, lesion level (paraplegia or tetraplegia) and time since injury. Further details regarding socio demographic or lesion characteristics, including completeness of lesion, etiology of SCI or center of first rehabilitation were not commonly available.

### Statistical analyses

We use the overall term participation rate to describe different types of rate calculations related to recruitment outcomes including contact, cooperation, response, and attrition rate [23]. The simplest approach to response rate calculation is to divide the number of participants by the number of invited subjects. However, this does not account for factors that can affect the response rate such as undelivered questionnaires or ineligibility of subjects who completed questionnaires. Typically, the numerator and denominator of the response proportion were adjusted to reflect such factors. According to internationally established guidelines [14, 23], we display four types of absolute (per specific module) and cumulative (up to a specific module) participation rates, i.e. the cooperation, contact, response and attrition rates (Table 1).

**Table 1:** The calculation of participation rates according to the American Association of Public Opinion Research, AAPOR [23]

	Abbreviations/Calculation	Explanation
Response status		
Participation	I	Subjects who participated in a module
Refusal	R	Subjects who refused to participate in a module
No contact	NC	Subjects who received the postal invitation but could not be personally contacted due to unavailability of telephone number or unsuccessful telephone calls (10 failed attempts); deceased or moved away from Switzerland after selection but before contact could be made
Not eligible	NE	Subjects who were excluded after first contact as they did not meet inclusion criteria defined in the study protocol (deceased or moved away from Switzerland before the date of the invitational letter; insufficient language skills in German, French or Italian; other medical diagnoses than defined in the study protocol; poor cognitive health status; non-existent or wrong contact information^a^)
Eligible	E = I + R + NC	Subjects eligible for the survey
Participation rates		
Absolute cooperation rate	[I/(I + R)]*100	% of subjects who participated of those who were contacted in a specific module
Cumulative cooperation rate	[I/(I + R_tot_)]*100	% of subjects who participated of those who were contacted up to a specific module
Absolute contact rate	[(I + R)/(I + R + NC)]*100	% of eligible subjects who could be contacted in a specific module
Cumulative contact rate	[(I + R_tot_)/(I + R_tot_ + NC_tot_)]*100	% of eligible subjects who could be contacted up to a specific module
Absolute response rate^b^	[I/(I + R + NC)]*100	% of subjects who participated of the total number of eligible subjects in a specific module
Cumulative response rate	[I/(I + R_tot_ + NC_tot_)]*100	% of subjects who participated of the total number of eligible subjects up to a specific module
Absolute attrition rate	[1- [I/(I + R + NC)]]*100	% of subjects who refused participation in a specific wave after participation in a former module
Cumulative attrition rate	[1- (I/(I + R_tot_ + NC_tot_))]*100	% of all subjects who refused participation after participation in a former module

^a^According to AAPOR’s definition, the category ‘non-existent or wrong contact information’ is grouped as ‘unknown if household/occupied household unit’ (UH), or ‘unknown, other’ (UO). For the 88 cases that never received the invitational letter, we carefully investigate their status with Swiss public authorities and could not detect their actual stay. We therefore assume that they are either deceased or moved away from Switzerland and categorized them as NE

^b^According to AAPOR’s definition ‘Response Rate 5’ assuming that there are no eligible cases among the cases of unknown eligibility (see footnote a)

The first two modules of the SwiSCI study population were compared in terms of differences in sociodemographic and lesion characteristics. Confidence intervals for the difference between modules were calculated using approximately normal distributed estimates for dependent, overlapping groups. In order to assess the achievement of the different recruitment efforts (invitation letter, first reminder, second reminder, telephone reminder), absolute, relative and cumulative response rates for each recruitment effort were computed.

To evaluate the existence of non-response bias in the principal module of the survey (second module) we used descriptive statistics to evaluate the distribution of available parameters characterizing the eligible population addressed by the survey (*n* = 3144) with those of participants in the second module (*n* = 1549). In this manuscript, the non-response analysis was performed only for the second module as the presented analyses are based on data from the second module which comprises the essence of the ICF-based questionnaire on functioning [8]. As reported elsewhere, the extent of cumulative non-response bias is essentially established in the second module and remains stable in third modules [24]. Following recommendation of the International Spinal Cord Society (ISCoS) [25], we used categorical variables for current age and time since injury for non-response analysis. Current age (in years) was thus categorized in classes 16–30, 31–45, 46–60, 61–75, and ≥76. Time since injury was classified as <6 years, 6–15 years, 16–25 years, and ≥26 years. Differential in odds of response were further evaluated using logistic regression analysis using likelihood ratio tests as global tests for variable significance. In case of test significance for parameters with more than two levels (i.e., age class; preferred language; time since injury), meaningful differences across levels were evaluated using pairwise comparisons with Bonferroni-adjustment as to account for multiple comparisons. Result are presented as forest plot displaying odds ratios (OR) with 95 % confidence intervals (CI).

Next, to test the association between responders characteristics and response speed, we applied ordinal logistic regressions using the 4-categorical variable on response speed to the second module as ordinal outcome (response after invitational letter, first reminder, second reminder, telephone reminder). It was not reasonable to use the linear variable ‘days until response’ as outcome as the sending of reminders may varied by a few days between participants due to feasibility reasons. The parallel lines assumption as prerequisite to apply ordinal logistic regression was confirmed with Stata’s gologit2 command and its autofit option with p-values using Brant tests for a single imputation [26]. To test the association between responders characteristics and response mode in the second module, we performed logistic regressions using a dichotomous outcome (online vs. paper-pencil). For both regression analyses on response behaviour, we report unadjusted and adjusted OR, its 95 % CI, and *p* values from Equal Fraction Missing information (FMI) tests.

To address the potential bias due to item non-response in the analyses on response behaviour and responders characteristics, we additionally carried out multiple imputations. More specifically, we used multiple imputation by chained equations (MICE) [27] enabling to impute different types of variables, including categorical, ordinal and linear variables. We incorporated all covariates of interest into the imputation model (except outcome variables as there were no missing values). For each model, ten imputations were carried out. Since results of analyses based on imputed datasets were comparable to the results found in complete case analyses, we only show results based on imputed data.

All analyses were conducted using STATA Version 13.1 for Windows (StataCorp, College Station, TX, USA).

## Results

As displayed in Table 2, sociodemographic and lesion characteristics of the SwiSCI study population were comparable between the first two modules of the survey.

**Table 2:** Basic characteristics of the SwiSCI study population

	First module	Second module	Difference
[Missing values first/second module]	*N* (%) or mean (SD); median (IQR)	*N* (%) or mean (SD); median (IQR)	Difference in % or mean (95 % CI)
Total	1922 (100)	1549 (100)	
Sociodemographic characteristics			
Male gender [0 missing values]	1376 (71.6)	1107 (71.5)	0.13 (0.12 to 0.14)
Age in years [0]	52.6 (15.3); 52 (42–64)	52.3 (14.8); 52 (42–63)	0.25 (−0.12 to 0.61)
Years of education [54/32]	13.4 (3.4); 13 (12–15)	13.6 (3.3); 13 (12–15)	−0.18 (−0.26 to −0.11)
Net equivalence household income in Swiss Francs [−/168]	Not assessed	4135.7 (1809.6),	
		3750 (2500–5250)	
Paid employment^a^ [79/68]	728 (39.5)	627 (42.3)	−2.84 (−2.85 to 2.83)
Living alone [−/41]	Not assessed	418 (27.7)	
Having a partner [79/62]	1210 (65.7)	1004 (67.5)	−1.86 (−1.88 to −1.85)
Language region [0]			
German	1379 (71.8)	1088 (70.2)	0.41 (0.40 to 0.42)
French	462 (24.0)	391 (25.3)	−0.24 (−0.25 to −0.23)
Italian	81 (4.2)	70 (4.5)	−0.18 (0.18 to −0.17)
Membership in SPA [0]	1324 (68.9)	1101 (71.1)	−2.19 (−2.20 to −2.18)
Lesion characteristics			
Lesion level [23/12]			
Paraplegia	1307 (68.8)	1063 (69.2)	−0.34 (−0.35 to −0.32)
Tetraplegia	592 (31.2)	474 (30.8)	0.34 (0.32 to 0.35)
Extent of lesion [16/9]			
Complete lesion	781 (41.0)	646 (42.0)	−0.97 (−0.98 to −0.96)
Incomplete lesion	1125 (59.0)	894 (58.1)	0.97 (0.96 to 0.98)
Aetiology [19/15]			
Traumatic	1491 (78.4)	1202 (78.4)	−0.01 (−0.02 to 0.00)
Non-traumatic	412 (21.7)	332 (21.6)	0.01 (0.00 to 0.02)
Years since injury [40/34]	16.6 (12.4); 13.5 (6–24)	16.7 (12.7); 13 (6–25)	−0.22 (−0.48 to 0.03)

Missing values were ignored when calculating percentages

*Abbreviations: SD* standard deviation, *IQR* interquartile range, *CI* confidence interval, *SPA* Swiss Paraplegic Association

^a^Includes also retired persons

### Objective 1: Description of participation rates

Eligibility, response status and participation rates for each module are presented in Table 3. In total, 3807 persons were invited for survey participation. A total of 663 persons were identified as non-eligible (16.3 %), indicating a total number of 3144 eligible persons for the first two modules. Of the 663 non-eligible persons, 308 did not meet inclusion criteria (*n* = 147 other medical diagnosis than defined in the study protocol; *n* = 117 not Swiss residents; *n* = 16 insufficient language skills; *n* = 28, other reasons), 50 were contacted by more than one institution (double contacts), 217 deceased before invitation and 88 could not be contacted as the postal mail was undeliverable (Table 3).

**Table 3:** Description of eligibility, response status and participation rates for all SwiSCI modules

	Abbreviations/Calculation	First module	Second module	Third modules		
				PPF-HB module	HSR module	Work module
Total invited	I + R + NC + NE	3807	1922	583	587	383
Eligibility, *n*						
Eligible total	I + R + NC = E_tot_	3144	3144	N.a.	N.a.	N.a.
Eligible for module	E_module_	3144	1922	572	579	377
Not eligible	NE	663	(55)^a^	(12)^a^	(9)^a^	(7)^a^
*Double contact*		*50*	*(12)*	*(1)*	*(1)*	*(2)*
*Deceased before invitation*		*217*	*N.a.*	*N.a.*	*N.a.*	*N.a.*
*Inclusion criteria not met*		*308*	*(43)*	*(11)*	*(8)*	*(5)*
*Undeliverable* ^*b*^		*88*	*0*	*0*	*0*	*0*
Response status, *n*						
Participation	I	1922	1549	511	492	328
Refusal	R	845	306	59	79	46
No contact	NC	377	67	1	7	2
*Not reached by telephone*		*191*	*32*	*0*	*0*	*0*
*No telephone number*		*179*	*22*	*0*	*0*	*0*
*Deceased after sent out*		*7*	*13*	*1*	*7*	*2*
Participation rates (%)						
Absolute cooperation rate	[I/(I + R)]*100	69.5	83.5	89.6	86.2	87.7
Cumulative cooperation rate	[I/(I + R_tot_)]*100	N.a.	57.4	For all 3^rd^ modules: 49.9		
Absolute contact rate	[(I + R)/(I + R + NC)]*100	88.0	96.5	99.8	98.8	99.5
Cumulative contact rate	[(I + R_tot_)/(I + R_tot_ + NC_tot_)]*100	N.a.	85.9	For all 3^rd^ modules: 85.4		
Absolute response rate^c^	[I/(I + R + NC)]*100	61.1	80.6	89.5	85.1	87.2
Cumulative response rate	[I/(I + R_tot_ + NC_tot_)]*100	N.a.	49.3	For all 3^rd^ modules: 42.7		
Absolute attrition rate	[1- [I/(I + R + NC)]]*100	38.9	19.4	10.5	14.9	12.8
Cumulative attrition rate	[1- (I/(I + R_tot_ + NC_tot_))]*100	N.a.	50.7	For all 3^rd^ modules: 57.4		

Absolute rates always focus on the numbers of a specific module

*Abbreviations: PPF-HB* Psychological Personal Factors and Health Behavior Module, *HSR* Health Services Research, *N.a.* not applicable, *tot* all modules up to the specific module

^a^Already included in the 663 NE from first module, otherwise participation rates would be biased

^b^See footnote a Table 1

^c^Calculation of response rate according to AAPOR’s definition ‘Response Rate 5’ assuming that there are no eligible cases among the cases of unknown eligibility (see footnote a Table 1)

Absolute cooperation rate was 69.5 % for the first module and between 83.5 and 89.6 % for subsequent modules. Cumulatively, nearly 50 % of all eligible persons who could be contacted completed all three modules (cumulative cooperation rate 49.9 % ). 12 % of eligible persons could not be contacted within the first module, however, absolute contact rates for subsequent modules were high (over 96.5 %). 61.1 % of all eligible persons participated in the first module, 80.6 % in the second, and between 85.1 and 89.5 % in the third modules (absolute response rates), indicating that around four out of five persons participated in the second module after having completed the first module. Cumulatively, around half of eligible subjects (49.3 %) completed the first two modules and 42.7 % of persons completed all three modules (cumulative response rates).

### Objective 2: Illustration of response rates in relation to recruitment efforts

**Fig. 2:**
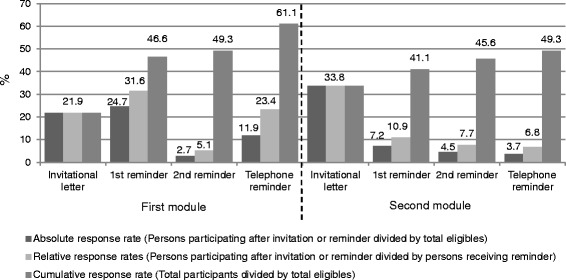
Absolute, relative and cumulative response rates of the first (*n* = 1922) and the second module (*n* = 1549) by recruitment efforts in %

Absolute, relative and cumulative response rates by recruitment efforts for the first two modules are displayed in Fig. 2. Response to the invitational letter was over 10 % lower in the first compared to the second module, potentially due to the fact that not interested persons already dropped during the first module. Overall, the first reminder in the first module showed considerable effects and reached even higher absolute response rates than the invitation, whereas the first reminder in the second module showed smaller effects on participation. The response to the second reminder was modest for both modules. Around one out of four persons having received a telephone reminder in the first module participated, but only one out of 15 participated after the telephone reminder in the second module. 202 persons directly responded to the first module by telephone interview. As the second module was much more comprehensive, telephone reminders were used to encourage subjects to complete the questionnaire. Considering the high time and financial expenses involved in telephone interviewing, this mode of response pertained only to 16 persons responding to the second module who were not able to fill in the questionnaire by themselves (e.g., due to limited hand function).

### Objective 3: Comparison between respondents and non-respondents

**Fig. 3:**
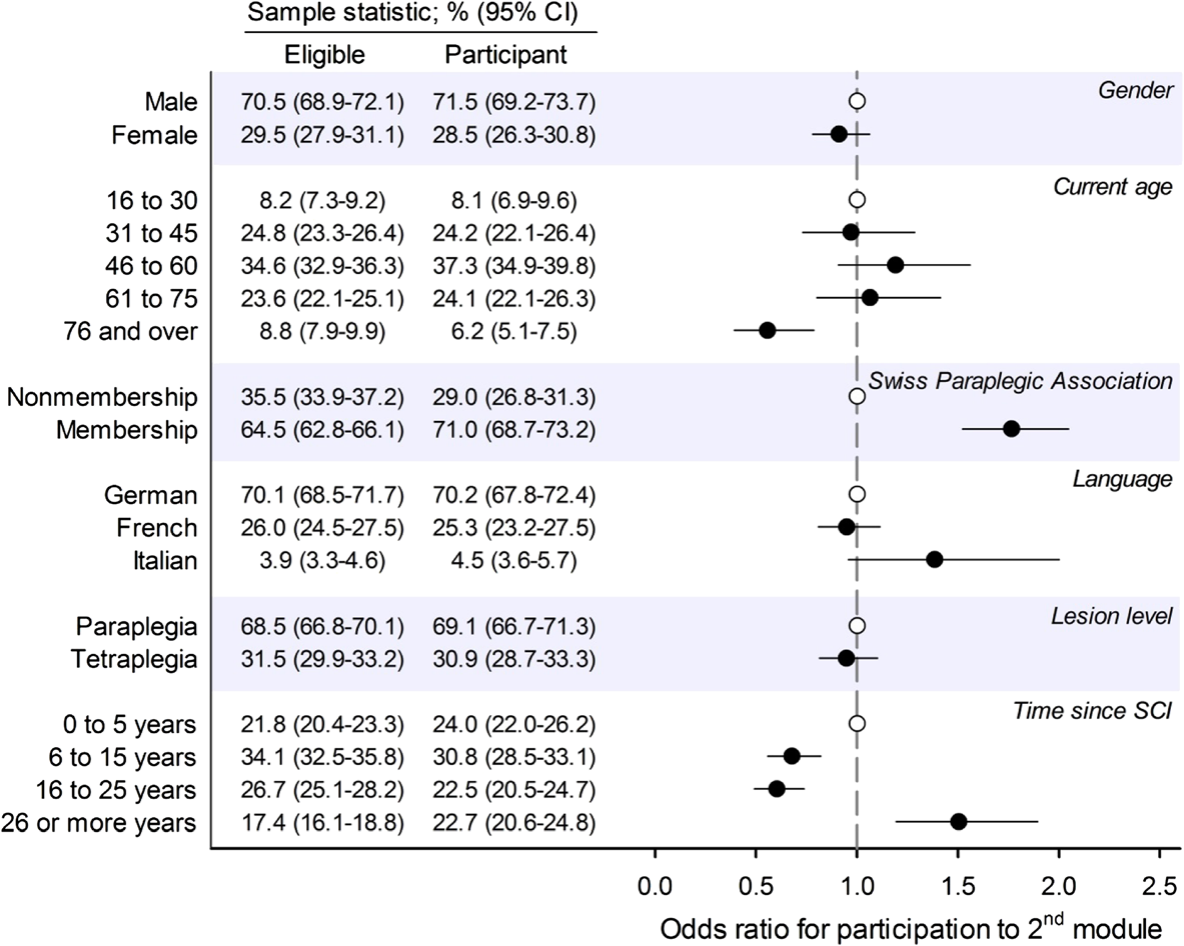
Non-response analysis

Figure 3 summarizes participation to the second module of the survey in relation to sociodemographic and lesion characteristics that were available for the analysis among all eligible subjects. Comparison of sample statistics of the eligible population (*n* = 3144) with the participant population to the second module (*n* = 1549) indicated differential rates and odds of participation across age groups (logistic regression, *p* < 0.001), with membership of the SPA (*p* < 0.001) and across time since SCI groups (*p* < 0.001). Members of the SPA were 1.7 times more likely to participate than non-members. Participation was diminished in persons aged ≥76 as compared to the all other age groups (post-hoc pairwise comparisons, all *p* < 0.01), while other age groups showed similar odds of participation (all *p* values >0.2). Finally, groups living with SCI for 6 to 15 years or 16 to 25 years showed relatively low odds of participation (all *p*-values < 0.001), while those living with SCI for ≥26 years showed relatively high odds (all *p*-values < 0.01).

### Objective 4: Description of response behaviour (speed and mode) in relation to responders’ characteristics

After mutually adjusting effect sizes for the effect of other parameters, only household income remained significantly associated with response speed, indicating that those having high income were less often reminded until participation (OR 0.89; 95 % CI 0.83 to 0.95). Sociodemographic or lesion characteristics, as well as indicators of health and functioning did not affect response speed (Table 4). The model assessment for proportional odds assumption indicated no violation (Brant test *p*-values between 0.08 and 0.95 for unadjusted, and 0.06 to 0.97 for adjusted analyses, respectively; 0.86 overall). No collinearity was found (variance inflation factor maximally 1.8, average 1.34). The adjusted model showed a predictive power of R^2^ for count data of 0.70 on a single imputation.

**Table 4:** Responders’ characteristics and response speed

	Model 1		Model 2	
	OR (95 % CI)	*p*	OR (95 % CI)	*p*
Sociodemographic characteristics				
Gender (Male)	Reference	0.803	Reference	0.379
Female	1.03 (0.81 to 1.30)		1.12 (0.87 to 1.43)	
Age (per 10 years)	0.92 (0.86 to 0.99)	0.023	0.94 (0.86 to 1.02)	0.139
Education (per year)	0.96 (0.93 to 0.99)	0.016	0.72 (0.93 to 1.00)	0.068
Household income (per 1000 Swiss Francs)	0.89 (0.84 to 0.95)	<0.001	0.89 (0.83 to 0.95)	<0.001
Employment (Yes)	Reference	0.468	Reference	0.078
No	1.09 (0.87 to 1.35)		1.28 (0.97 to 1.69)	
Living arrangement (With others)	Reference	0.025	Reference	0.168
Living alone	1.31 (1.03 to 1.65)		1.24 (0.91 to 1.69)	
Partnership (Having a partner)	Reference	0.007	Reference	0.192
Not having a partner	1.37 (1.09 to 1.71)		1.22 (0.91 to 1.64)	
Language (German)	Reference	0.645	Reference	0.619
French	0.95 (0.74 to 1.21)		0.87 (0.67 to 1.12)	
Italian	1.21 (0.74 to 1.95)		1.09 (0.96 to 1.78)	
Membership Swiss Paraplegic Association (Non-member)	Reference	0.675	Reference	0.915
Member	0.95 (0.76 to 1.20)		1.01 (0.78 to 1.32)	
Lesion characteristics				
Lesion level (Paraplegia)	Reference	0.208	Reference	0.532
Tetraplegia	1.16 (0.92 to 1.45)		1.08 (0.84 to 1.39)	
Extent of lesion (Complete)	Reference	0.051	Reference	0.076
Incomplete lesion	1.23 (1.00 to 1.54)		1.24 (0.98 to 1.60)	
Aetiology (Traumatic)	Reference	0.873	Reference	0.744
Non-traumatic	0.98 (0.76 to 1.26)		0.95 (0.72 to 1.27)	
Time since injury (per 10 years)	0.99 (0.99 to 1.00)	0.136	1.00 (0.99 to 1.01)	0.841
Health & functioning indicators				
Quality of life (0–4; per point)	1.10 (0.77 to 1.06)	0.225	0.97 (0.81 to 1.18)	0.783
Mental health (0–100; per 10 points)	0.94 (0.81 to 1.09)	0.400	0.97 (0.83 to 1.12)	0.657
Secondary conditions (0–39; per 10 points)	0.97 (0.92 to 1.01)	0.591	0.94 (0.88 to 1.00)	0.347
Participation (0–100; per 10 points)	0.95 (0.78 to 1.14)	0.166	0.89 (0.70 to 1.13)	0.053

Unadjusted and adjusted odds ratios (OR) and (95 % confidence intervals, CI) from ordered logistic regression on response speed in the second module, OR above 1.00 indicate more reminders until participation. For each parameter, the reference category (for group variables) or the reference scale (for continuous variables) is indicated in parenthesis. *p* values from Equal Fraction-Missing-Information (FMI) test. Results from analysis with multiple imputed datasets, *n* = 1549. Model 1: unadjusted. Model 2: adjusted for all covariates

**Fig. 4:**
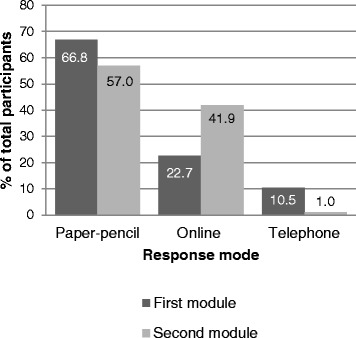
Response modes in the first (*n* = 1922) and the second module (*n* = 1549) in %

Participants showed an overall preference to complete the paper-pencil version of the questionnaire, and particularly so for the brief first module; for completing the subsequent and lengthier second module, 41.9 % opted for online completion. Among this proportion of online completers, 3.7 % of persons who started online completion have interrupted the questionnaire somewhere before the last 20 items. Telephone interviews were less often performed in the second module (Fig. 4). After adjustment for confounding, male gender, younger age, higher education, higher income, and SPA membership were significantly associated with online completion. Persons with tetraplegia, longer time since injury, higher quality of life and more participation restrictions also completed online more often (Table 5). The model showed low predictive power (McFadden R^2^ = 0.1, on single imputation) and low collinearity between predictor variables (variance inflation factor maximally 1.54; 1.31 in average).

**Table 5:** Responders’ characteristics and online response

	Model 1		Model 2	
	OR (95 % CI)	*p*	OR (95 % CI)	*p*
Sociodemographic characteristics				
Gender (Male)	Reference	<0.001	Reference	<0.001
Female	0.47 (0.37 to 0.59)		0.47 (0.37 to 0.59)	
Age (per 10 years)	0.73 (0.68 to 0.78)	<0.001	0.70 (0.64 to 0.77)	<0.001
Education (per year)	1.11 (1.08 to 1.15)	<0.001	1.07 (1.03 to 1.11)	<0.001
Household income (per 1000 Swiss Francs)	1.17 (1.10 to 1.24)	<0.001	1.10 (1.03 to 1.18)	0.008
Employment (Yes)	Reference	<0.001	Reference	0.300
No	0.55 (0.44 to 0.68)		0.87 (0.66 to 1.14)	
Living arrangement (With others)	Reference	0.770	Reference	0.864
Living alone	0.97 (0.77 to 1.22)		1.02 (0.73 to 1.44)	
Partnership (Having a partner)	Reference	0.997	Reference	0.600
Not having a partner	1.00 (0.80 to 1.24)		0.92 (0.66 to 1.27)	
Language (German)	Reference	0.051	Reference	0.160
French	0.76 (0.60 to 0.96)		0.76 (0.55 to 1.03)	
Italian	0.76 (0.46 to 1.25)		0.74 (0.39 to 1.38)	
Membership Swiss Paraplegic Association (Non-member)	Reference	<0.001	Reference	0.006
Member	2.27 (1.84 to 2.97)		1.48 (1.12 to 1.96)	
Lesion characteristics				
Lesion level (Paraplegia)	Reference	<0.001	Reference	<0.001
Tetraplegia	1.94 (1.55 to 2.42)		1.63 (1.26 to 2.12)	
Extent of lesion (Complete)	Reference	<0.001	Reference	0.757
Incomplete lesion	0.65 (0.53 to 0.80)		0.96 (0.75 to 1.24)	
Aetiology (Traumatic)	Reference	<0.001	Reference	0.978
Non-traumatic	0.51 (0.39 to 0.66)		0.99 (0.73 to 1.36)	
Time since injury (per 10 years)	1.11 (1.02 to 1.20)	0.015	1.14 (1.03 to 1.26)	0.010
Health & functioning indicators				
Quality of life (0–4; per point)	1.30 (1.12 to 1.52)	<0.001	1.28 (1.05 to 1.56)	0.013
Mental health (0–100; per 10 points)	0.98 (0.85 to 1.12)	0.734	0.89 (0.76 to 1.04)	0.147
Secondary conditions (0–39; per 10 points)	1.05 (0.88 to 1.26)	0.561	1.04 (0.84 to 1.30)	0.709
Participation (0–100; per 10 points)	0.93 (0.89 to 0.98)	0.003	0.85 (0.79 to 0.91)	<0.001

Unadjusted and adjusted odds ratios (OR) and (95 % confidence intervals, CI) from binary logistic regression for online response in the second module. *p* values from Equal Fraction-Missing-Information (FMI) test. Results from analysis with multiple imputed datasets, *n* = 1533 (16 cases with telephone interviews were excluded). Model 1: unadjusted. Model 2: adjusted for all covariates

## Discussion

We have reported on the recruitment strategy, the participation rates, the non-response bias and the response behaviour in the SwiSCI community survey, the largest cohort on persons with SCI in Europe. Over the successive modules, absolute response rates were 61.1, 80.6 and 87.3 % which resulted in cumulative response rates of 49.3 and 42.6 % for the second and third modules. We found that a first written reminder was valuable in increasing the response, while the second written reminder did not enhance response considerably. The telephone reminders were useful within the first module where people were directly interviewed by telephone but its effects were limited in the subsequent modules where a telephone interview was not explicitly offered due to the length of the questionnaires. Non-response bias was minor in this survey since gender, preferred language and lesion level were not associated with participation. However, membership of SPA, age and time since injury were associated with study participation. Furthermore, household income was the only factor related to response speed, while response mode (paper-pencil vs. online) differed according to participants’ characteristics.

Although there is no agreed-upon standard for acceptable response rates [28, 29], a response rate of 60 % has long been accepted as threshold of sufficient participation [29]. With respect to these recommendations, we have reached a good response rate in the first module, whereas the response to the subsequent modules would be classified as insufficient. However, graded recommendations for different waves of data collection are needed and thresholds might be set higher to allow for reasonable attrition rates. Furthermore, thresholds for response rates are only a rule of thumb that do not necessarily cover a far more important criterion of survey quality, namely the bias introduced by non-response. There is increasing evidence that a survey’s response rate may not be as strongly associated with quality or representativeness as postulated in the past [29]. Although we found that survey participation is associated with membership of SPA, time since injury and age, the quality of our sample can be rated as satisfactory as a separate paper documented that the non-response bias did not significantly affect key outcomes of the study [24]. Furthermore, we could not detect non-response bias concerning gender, language and lesion level, i.e. the severity of disability did not seem to influence study participation.

Comparable to other surveys using the strategy of repeated mailings following initial non-response [30, 31], we could increase response rates considerably during the first module (40 % increase after initial invitation), however, effects were somehow lower in subsequent modules (e.g. 16 % in the second module), potentially also because of the length of the second and the third modules. Although evidence showed that mailed surveys have generally higher responses than online surveys [32, 33], we believe that it is important to offer both modes, paper-pencil and online, as we found that personal and lesion characteristics are associated with the preferences in response modes. However, this recommendation might not be generalizable for surveys in general populations as a meta-analysis revealed that offering web-based questionnaires simultaneously with mailed surveys might be less effective than offering paper-pencil mode exclusively [34]. In the case of the SwiSCI community survey, it is nevertheless of primordial importance to offer both modes as some participants encounter difficulties with handwriting and rely on the use of assistive devices to operate computers. Also, impaired mobility is obviously far more common in SCI populations and likely to reduce survey response when only providing the option of questionnaire return by postal mail. Still, as we did not use an experimental design to test effectiveness of applied methods, we cannot conclude that response rates would have been lower if another response scheme would have been offered (e.g. only paper-pencil or only online; telephone interviews for all modules). Given the fact that many persons receiving a telephone reminder in the first module agreed on doing a telephone interview, we would recommend to offer telephone interviews also for longer surveys whenever resources are available. However, in a high resource country such as Switzerland, the assumption is justified that most persons with disabilities have access to personal assistive devices that support them with writing and/or using a telephone or computer. Indeed, we do not have information on the use of such devices and can therefore not conclude that some persons refused participation due to a lack of assistive devices.

Household income was the only factor that was associated with response speed defined by how many reminders one has received until participation. Although it is surprising that no other factors were associated with response speed, this finding is in line with a large body of evidence stating that survey participation positively correlates with socioeconomic position [35]. The rate of online completion increased with increasing length of the questionnaire and varied considerably by specific responder characteristics. In accordance with data on internet use, males, younger persons and persons from higher socioeconomic groups preferred online completion [36]. The finding that persons with tetraplegia, longer time since injury and persons with more participation restrictions used the online version more often is not surprising, as these people may use assistive technologies and therefore could overcome potential difficulties with fine hand use.

Due to the fact that recruitment was based on lists of cooperating clinics and SCI associations, many invited persons were registered in lists with outdated information, for example, around 7 % of invited persons have deceased before invitation. Also, almost 5 % of invited subjects had another diagnosis than defined as inclusion criteria in the study protocol. Patient associations oftentimes include persons with a broader disability-spectrum and do not dispose on specific information on medical diagnosis needed for the assessment of study eligibility. Another particular challenge of the SwiSCI survey was to obtain a comprehensive and detailed picture of the participants’ situation while keeping the questionnaire in acceptable length to minimize drop-out and response bias [37].

Several limitations have to be considered. First, although we included three specialized rehabilitation centres and the most important associations for persons with SCI (SPA) , there are uncertainties in relation to the total SCI population in Switzerland. Second, information on the assistance from third persons or the use of assistive devices for study participation (e.g. assistive technologies to facilitate online completion in tetraplegics) is missing. Therefore, we cannot assess whether a lack of personal support or assistive devices enhanced non-response or not. Third, as we do not use an experimental design, we cannot conclude that the used methodologies have advantages over other procedures. Fourth, self-report data might be susceptible to reporting bias such as socially desired responding or recall bias. We have shown elsewhere that self-reported information on demographics and lesion characteristics showed good consistency with available medical records data [24], but cannot exclude self-report bias on other information such as income or health conditions. These limitations are balanced by several strengths. First, we were able to recruit one of the largest population-based samples of persons with SCI in Europe and could show that non-response bias is marginal in this sample. Second, we dispose on a large and detailed database on response behaviour and third, we used currently recommended guidelines to transparently report participation rates.

## Conclusion

Given the fact that surveying persons with disabilities is especially challenging, achieved response rates of the SwiSCI community survey were satisfying. In line with current survey methodology [10], the recurrent mixed-mode reminding might be a key feature of optimizing survey design in a study population facing impairment or disability as, for example, the first written reminder increased response up to 40 % in the first module. Since we found large effects of telephone reminders in the first module where persons could directly opt for a telephone interview, we would recommend to offer telephone interviews also for longer surveys whenever resources are available. Although there are hints that offering paper-pencil and online versions simultaneously might be less effective [34], we would recommend to offer both response options in a population with potential impairments in hand functions and mobility limitations.
